# Nanoparticles for Enhanced Adoptive T Cell Therapies and Future Perspectives for CNS Tumors

**DOI:** 10.3389/fimmu.2021.600659

**Published:** 2021-03-23

**Authors:** Preethi Bala Balakrishnan, Elizabeth E. Sweeney

**Affiliations:** The George Washington University Cancer Center, School of Medicine and Health Sciences, George Washington University, Washington, DC, United States

**Keywords:** nanoparticles, nanotechnology, T cell therapy, immunoengineering, CNS tumors, solid tumors, artificial antigen-presenting cells, adoptive cell therapy

## Abstract

Adoptive T cell therapy has emerged as a revolutionary immunotherapy for treating cancer. Despite immense promise and clinical success in some hematologic malignancies, limitations remain that thwart its efficacy in solid tumors. Particularly in tumors of the central nervous system (CNS), T cell therapy is often restricted by the difficulty in intratumoral delivery across anatomical niches, suboptimal T cell specificity or activation, and intratumoral T cell dysfunction due to immunosuppressive tumor microenvironments (TMEs). Nanoparticles may offer several advantages to overcome these limitations of T cell therapy, as they can be designed to robustly and specifically activate T cells *ex vivo* prior to adoptive transfer, to encapsulate T cell stimulating agents for co-localized stimulation, and to be conjugated onto T cells for added functionality. This perspective highlights recent preclinical advances in using nanoparticles to enhance T cell therapy, and discusses the potential applicability and constraints of nanoparticle-enhanced T cells as a new platform for treating CNS tumors.

## Introduction

Adoptive T cell therapy, including antigen-specific T cells and chimeric antigen receptor (CAR) T cells, represents a promising avenue for treating cancer, as evidenced by numerous clinical trials in hematologic and solid tumors in both the adult and pediatric populations (1–7). Indeed, three T cell-based therapeutics, Kymriah, Yescarta, and Breyanzi have been approved by the US FDA for treating B cell leukemia and lymphoma (8–10). Despite immense potential in hematologic malignancies, T cell therapy inadequately controls tumor growth in many solid tumor contexts due to intrinsic limitations (11–14).

Firstly, effective T cell therapy can be hindered by the heterogeneous tumor antigen milieu, such that T cells engineered to target only one antigen may be ineffective in clearing the tumor. Abnormal blood vessels, typical of solid tumors (15), hinder T cell infiltration into the tumor microenvironment (TME), thus limiting their local bioavailability and function. Additionally, the TME of solid tumors is typically immunosuppressive. Thus, even when T cells are able to infiltrate and target the heterogeneous tumor-associated antigens, the TME signals suppression of T cell function, thereby blocking their therapeutic effect (16). Tumors of the central nervous system (CNS) are especially difficult to treat with T cell therapy, due to the increased difficulty of T cells to traffic to and infiltrate CNS anatomical regions, thereby dampening their effect (17, 18).

Nanotechnology offers advantages to overcome limitations of T cell therapy for solid tumors. Because of their size, surface area to volume ratio, and ability to encapsulate a variety of agents for controlled release, nanoparticles have long been studied for their advantages in drug delivery in cancer (19, 20). Nanoparticles offer a means to cross various anatomical niches, such as the blood brain barrier (BBB), to deliver therapeutic cargo, enabling both imaging and therapy of solid tumors. Over the past several decades, many nanoparticles have been explored and validated in preclinical brain tumor models (21–33). There is emerging research into how nanotechnology can be used to improve immunotherapies, including T cell therapy (34–38), but very few studies, if any, investigate the role to nanoparticles to improve T cell therapy for CNS tumors. Here, we aim to summarize the recent developments in nanoparticle-enhanced T cell therapy, and comment on hypothesized applications for treating CNS tumors. We highlight nanoparticle-mediated strategies to prepare T cell products for adoptive therapy, nanoparticles conjugated to T cells to overcome TME immunosuppression, and nanoparticles to enhance T cell tumor infiltration, activate T cells *in situ*, and/or add functionality to T cell therapy. Finally, we discuss how to apply the described principles and strategies of nanoparticle-enhanced T cells to treat tumors of the CNS.

## Nanoparticles to Improve the *Ex Vivo* Generation of Therapeutic T Cell Products

### Nanoparticles for Improving T Cell Manufacture

To improve *ex vivo* expansion of antigen-specific T cells, one group generated a nanostructured polyethylene glycol (PEG) hydrogel platform to stimulate T cells prior to adoptive transfer (39). The presented platform used gold nanoparticles conjugated with anti-CD3 antibodies to activate T cells, and integrin-activating peptides to initiate integrin-mediated cell adhesion of the hydrogel to T cells. Overall, the nanostructured hydrogel enabled T cell activation, proliferation, and memory (39).

In an effort to arm T cells to counter the immunosuppressive microenvironment typical of solid tumors, immunoliposomes were generated to encapsulate a small molecule TGF-β inhibitor (thereby avoiding toxic systemic administration of TGF-β blockade) and T cell targeting receptors CD45 or CD90 (40). The choice of the two receptors was to compare the effects of targeting a receptor likely to internalize the immunoliposomes (CD90) versus a receptor that would likely bind the immunoliposomes on the cell surface (CD45). These immunoliposomes enabled enhanced T cell activation and granzyme expression when incubated with T cells prior to adoptive transfer. Upon adoptive transfer, the T cells incubated with immunoliposomes targeting CD90 caused the most T cell-mediated anti-tumor activity and reduction in tumor growth *in vivo* in mice. These studies uncovered the importance of using an internalizing receptor (CD90) to target drug-loaded liposomes to T cells during manufacture (40).

Another strategy used small interfering RNA (siRNA) to downregulate immunosuppressive signaling pathways in the cytotoxic T cells before adoptive transfer, so as to improve their anti-tumor efficacy *in vivo* (41). Because the viability of primary T cells is sometimes affected by conventional or viral transduction or electroporation, the group attached gold nanoparticles to the T cells and used photoporation to gently heat the T cells and allow the membrane to transiently accept siRNA. They found successful gene silencing of those targeted by the siRNA, while maintaining the viability of the T cells (41). Significantly less T cell death occurred in response to transfection *via* the photoporation technique in comparison to traditionally employed nucleofection.

Polymeric nanocarriers that encapsulate mRNA have also been studied to transiently deliver mRNA to antigen-specific T cells prior to adoptive transfer (42). Researchers showed the ability of the nanocarriers to deliver mRNA to knock down immunosuppressive molecules in antigen-specific T cells, and induce transcriptional activity typical of a memory T cell phenotype. They illustrated the simple design of the strategy of mixing therapeutic T cells with the nanocarriers, and highlighted the broad applicability to overcoming various limitations of T cell therapy by targeting specific genes of interest (42).

Ionizable lipid nanoparticles can also mediate *ex vivo* mRNA delivery into T cells for transient CAR expression, thereby mitigating the toxic side effects seen in permanent CAR expression on T cells and circumventing classical mRNA delivery into cells (e.g. electroporation) which often impacts T cell viability (43). Indeed, a study showed effective CD19-specific CAR expression on T cells after lipid nanoparticle delivery of CD19 CAR mRNA, with decreased cell death as compared to electroporation-delivered CAR mRNA. Additionally, CAR T cells manufactured by this method *via* ionizable lipid nanoparticles induced functional protein expression and enabled anti-tumor efficacy in a leukemia model *in vitro* at equivalent levels to electroporated CAR T cells (43).

### Nanoparticles as Artificial Antigen-Presenting Cells (aAPCs)

*Ex vivo* expanded antigen-specific T cells generated using antigen-presenting cells (APCs) can specifically target tumor cells and exert effector functions (44, 45). Artificial antigen-presenting cells (aAPCs) represent a new class of nanoparticles to mimic T cell recognition and stimulation *ex vivo.* Several nanoparticle platforms are being investigated for improving the manufacture of T cells prior to adoptive transfer. An emerging area of interest is using nanoparticle-based aAPCs to increase the activation, specificity, and functionality of T cell products in various applications (46). Carbon nanotubes have been a focus of research in T cell manufacture, as the high surface area to volume ratio of single-walled carbon nanotubes allows high density of immobilized antigens and/or T cell activation molecules (47). Researchers showed the ability of carbon nanotubes to present high concentrations of anti-CD3 antibody to T cells and stimulate T cells more than free anti-CD3 or anti-CD3 bound to beads, illustrating their potential to generate activated T cells prior to therapy (48). Additionally, they found that when the single-walled carbon nanotubes were functionalized with anti-CD3 and other antibodies to co-stimulate T cells (anti-CD28), the high potency of T cell activation was driven by the concentration of bound antigens on the nanotubes (48). This strategy enabled rapid expansion of T cells for the purpose of adoptive therapy. A carbon nanotube-polymer composite with conjugated T cell stimuli (MHC-I and anti-CD28) has been used as an aAPC in the presence of IL-2. This nano-composite allowed effective T cell expansion with 1000x less IL-2 than standard T cell manufacture, and enabled delayed melanoma growth in mice (49).

Another group generated aAPCs by immobilizing a peptide from a model antigen, ovalbumin (OVA), loaded onto MHC-I dimers, along with anti-CD28, on biocompatible polymer-based biodegradable microparticles containing IL-2 (50). Upon co-culture of T cells with these aAPCs, T cells were able to more efficiently expand and activate compared to treatment with exogenous IL-2, aAPCs, or aAPCs in combination with exogenous IL-2, allowing for significant generation of OVA-specific cytotoxic T cells (50). Paramagnetic nanoparticles have also been utilized as aAPCs (nano-aAPCs) for improved T cell manufacture for adoptive transfer (51). Nano-aAPCs enriched tumor-specific T cells (targeting melanoma-associated antigens such as TRP2 and GP100), and enabled their rapid expansion both *in vitro* and *in vivo*. T cells generated *via* nano-aAPCs were also effectively stimulated upon antigen recognition, ultimately illustrating the potential of the platform to rapidly generate high amounts of tumor-specific T cells (51).

In addition to particles functioning as aAPCs, a group also investigated the use of a biomimetic scaffold to mimic APCs for increased T cell product output (52). A lipid bilayer comprising T cell-activating and co-stimulatory molecules (i.e. anti-CD3, anti-CD28) was combined with silica microrods for sustained release of cytokines (i.e. IL-2) to ensure sustained T cell function. They posited that the T cell product expansion can be tuned based on the structure and pattern of the scaffold and its encapsulated contents, and demonstrated the improved efficacy of the aAPC scaffold to efficiently expand polyclonal and antigen-specific (i.e. specific for peptides from Epstein-Barr virus (EBV)-associated proteins) cytotoxic T cells for use in adoptive therapy, as compared to traditional methods for T cell product generation. They illustrate the efficacy of the platform to produce CD19-specific CAR T cells as well, by illustrating effective lymphoma regression after adoptive therapy *in vivo* (52).

Another group investigated the use of biomimetic magnetosomes as aAPCs (53). The biomimetic magnetosomes comprised magnetic nanoclusters coated with immune cell membranes, anti-CD28 to stimulate T cells, and peptide-loaded MHC-I complexes, which allowed for robust *ex vivo* T cell expansion prior to adoptive transfer, as well as a means to physically guide the activated T cells by a magnetic field. Ultimately, the combination of the biomimetic nanoclusters and T cells enabled delayed lymphoma growth in mice *in vivo*, thus representing a platform to both improve therapeutic T cell generation while adding functionality (magnetically controlled tumor accumulation) *in vivo.*

## Nanoparticles to Overcome T Cell Immunosuppression in the TME

In addition to nanoparticle-mediated T cell engineering prior to adoptive transfer, other groups investigate the use of nanoparticles within the TME. Intratumoral T cell function is often dysregulated or inhibited in solid tumors, thereby preventing efficacy of adoptive T cell therapy (54–58). To overcome this, several groups have utilized nanoparticles loaded with various cargo aimed at maintaining T cell activation or blocking immunosuppressive pathway molecules. One potential solution is administering liposomes encapsulating T cell-activating antibodies or cytokines to target adopted T cells *in vivo* (59). Here, researchers used Thy1.1- and IL-2-conjugated liposomes to specifically target T cells and enable T cell proliferation, respectively (59). These studies illuminated the feasibility of effectively targeting T cells *in vivo* to deliver payloads (e.g. imaging agents, antibodies, cytokines, adjuvants, drugs) loaded within liposomes *via* surface targeting. Similarly, coating therapeutic T cells with nanoparticles loaded with adjuvant (i.e. IL-15 superagonist (IL-15SA) and IL-21) enabled adopted antigen-specific T cells to proliferate *in vivo* and effectively eliminate melanoma in a mouse model (60).

Another study illustrated a novel platform comprising T cells conjugated with nanogels loaded with proteins to enhance T cell function (61). Importantly, the protein cargo was designed to be released only upon antigen binding to T cell receptor, such that the likely activation site would be intratumoral. They found that when IL-15SA was encapsulated in the nanogel, transferred T cells had significantly higher expansion in tumors, and allowed effective CAR T cell-mediated tumor clearance *in vivo* (61). Alternatively, CAR T cells have been engineered to deliver liposomal nanoparticles encapsulating a small molecular adenosine antagonist in order to block the immunosuppressive function of adenosine within the TME of solid tumors that impairs T cell function (62). Because of the difficulty in delivering the antagonist specifically to T cells in the TME, the physical conjugation of the nanoparticles to the adoptive CAR T cells enables effective local delivery and provides a potential solution to overcoming an immunosuppressive pathway in solid tumors (62).

Other recent work has presented a novel nanoparticle platform for pre-treating the TME prior to CAR T cell therapy (63). There, lipid nanoparticles carrying a PI3K inhibitor were coated with a tumor-targeting peptide (iRGD). The nanoparticles also encapsulated α-GalCer, a component to activate NKT cells. The combination comprised in the nanoparticle platform was able to overcome the immunosuppressive TME that typically inhibits the success of CAR T cell therapy. Thus, CAR T cell therapy following nanoparticle treatment appeared successful in terms of T cell expansion and tumor regression (63). Other nanoparticles designed to remodel the TME could also play a role in improving the infiltration, activation, or functionality of adoptive T cell therapy (64).

These studies critically demonstrate the enhancement of T cell function through the use of conjugated nanoparticles. As nanoparticles can be designed to encapsulate innumerable disease- or cell-specific cargo, there is promise for vast applicability of platforms co-localizing nanoparticles and T cells.

## Nanoparticles to Aid T Cell Tumor Infiltration and *In Situ* T Cell Activation

To overcome some of the physical barriers of T cell infiltration in addition to the local immunosuppression in solid tumors, one group employed photothermally activated nanoparticles (PLGA nanoparticles encapsulating ICG) to enable mild hyperthermia in the TME (65). They showed that pre-treating melanoma tumors with nanoparticle-mediated photothermal therapy enabled significantly more CAR T cell accumulation and better anti-tumor efficacy *in vivo* (65). The photothermal therapy allowed physical disruption of the TME, increased blood flow, and decreased pressure, but also released tumor-specific antigens that could importantly stimulate the CAR T cells upon infiltration. Nanoparticle-mediated photothermal therapy is not a clinically approved treatment strategy, but there have been clinical trials that completed testing its efficacy in other applications (66), thus suggesting its future clinical potential.

Other researchers employed magnetic nanoclusters that were synthesized with anti-PD-1 antibodies in order to bind to PD-1 expressing T cells prior to adoptive transfer. Then, a commercial neodymium magnet was used to magnetically recruit the nanocluster-T cells to the site of tumor, overcoming barriers of tumor infiltration, where the structures disassembled, allowing the simultaneous anti-tumor effects of T cells and anti-PD-1 antibodies (67). Although anti-PD-1 antibodies are FDA-approved for several applications, magnetically-responsive nanoparticles are still currently under investigation in clinical trials (68).

Another interesting strategy to combat transferred T cell infiltration issues uses DNA nanocarriers to transfect CAR genes into endogenous T cells, thereby circumventing the limitations involved in T cell manufacture. A group demonstrated feasibility of this approach using polymer-based nanoparticles carrying leukemia-specific CARs, showing effective generation of CAR T cells *in vivo* and anti-tumor efficacy at equivalent levels to traditionally infused 194-1BBz-specific CAR T cells (69). Another group investigated the use of circular bispecific aptamers to bypass the *ex vivo* generation of antigen-specific T cells (70). The aptamer was designed to bind both naïve T cells and tumor cells, thereby activating the T cells *in situ.* This strategy allowed T cell accumulation in the TME and a local activation of aptamer-bound T cells. A variety of antigen-specific aptamers could be used in order to treat a broad range of tumors (70). These designs may eliminate the need for cell engineering prior to adoptive transfer, although their clinical application has not yet been reported.

## Nanoparticles to Add Functionality as Combination T Cell Therapy

Recently, researchers showed the potential of attaching Prussian blue nanoparticles enabling photothermal therapy to antigen-specific T cells for a combined nanoimmunotherapy (71). By combining disparate elements into a single platform, they demonstrated the improved efficacy of EBV-associated antigen-specific T cell function and nanoparticle-mediated photothermal therapy *in vitro* by increased target cell cytotoxicity (71). A different approach sought to combine the advantages of CAR T cells and nanoparticle-mediated photothermal therapy for hepatocellular carcinoma (72). IR780 nanoparticles were loaded on mesoporous silica, and subsequently coated with the membrane of CAR T cells specific to GPC3+ hepatocellular carcinoma. The CAR T cell membrane enabled the nanoparticles to target GPC3 on the surface of hepatocellular carcinoma cells *in vitro* and *in vivo* (72). This study represents a different approach to CAR T cell therapy, by isolating the functional component of CAR T cell targeting and adding it to a nanoparticle component. This work, however, relies on the photothermal capability of the nanoparticle to mediate cytotoxicity against tumor cells, rather than the cytotoxic functionality of CAR T cells, which could also introduce limitations, as the absence of T cells may reduce the generation of tumor-specific T cell expansion or T cell memory.

## Discussion

Thus far, we have summarized the state of the field of nanoparticle-enhanced therapeutic T cells for treating cancer. The unique attributes of nanoparticles, for example, their ability to penetrate anatomical barriers, encapsulate or immobilize therapeutic cargo, and specifically target tumor cells, add to their potential for enhancing T cell therapy (**Table 1**) (19, 20).

**Table 1:** Types of nanoparticles and their application in enhancing T cell therapy for solid tumors.

Nanoparticles	Function and Application in T cell Therapy	Ref
**Carbon Nanotubes**	High surface-to-volume ratio of nanotubes permits high-density coating of antigens/anti-CD3 and co-stimulatory molecules/cytokines for T cell activation	(47–49)
**Polymer-based particles**	Hollow center of polymeric shell can hold cytokines (e.g. IL-2) and help sustained release, reducing the effective concentration Gold nanoparticles coated with anti-CD3 antibodies were dispersed in an integrin peptide- crosslinked PEG hydrogel, creating a specific nanostructured surface, promoting integrin-mediated T cell binding and T cell activation and expansion Polymeric nanocarriers can promote transient delivery of mRNA to T cells to reverse immunosuppression and promote a T cell memory phenotype PLGA nanoparticles loaded with ICG for photothermal treatment induce mild hyperthermia at tumor site to improve CAR T cell penetration and tumor debulking	(39, 42, 50, 65)
**Magnetic nanoparticles**	anti-CD3- and anti-CD28-coated paramagnetic nanoparticles enable activation of T cells for weeks *ex vivo* Cell membrane-coated magnetosomes with conjugated anti-CD28 and anti-MHC-I can activate T cells and enable magnetic accumulation of T cells in the tumor and MRI imaging Magnetic nanoclusters carrying anti-PD-1 antibodies selectively target the TME by magnetic accumulation, and reverse immunosuppression prior to adoptive T cell transfer	(51, 53, 67)
**Lipid nanoparticles**	PEGylated immunoliposomes coated with anti-CD45 or anti-CD90 antibodies and loaded with TGF-β allow for T cell activation, granzyme production, and reversal of TME immunosuppression, while avoiding the systemic toxicity of free agents Ionizable liposomes carrying CAR mRNA transiently deliver CAR to T cells, and mitigate toxicity seen in permanent CAR expression on T cells, with improved functional protein expression and anti-tumor efficacy Thy1.1 and IL-2 conjugated liposomes allow for T cell targeting and selective delivery and improved T cell proliferation *in vivo* Maleimide-functionalized liposomes carrying IL-15SA and IL-21 conjugated to CD8+ T cells effect continuous pseudo-autocrine stimulation for adoptive T cell activation *in vivo* Liposomes loaded with A2aR-specific antagonists and attached to CAR T cells effect local delivery to overcome immunosuppression at TME caused by adenosine-binding to T cells, and rescues hypofunctional CAR T cells in TME Lipid nanoparticles coated with tumor-targeting peptides and loaded with PIK3 inhibitors selectively target tumor cells and reverse tumor immunosuppression prior to CAR T cell therapy	(40, 43, 59, 60, 62, 63)
**Gold Nanoparticles**	siRNA-coated gold nanoparticles can promote transient uptake (alternate to conventional viral transduction or electroporation) of siRNA by T cells for reversing immunosuppression and improving anti-tumor efficacy	(41)
**Biomimetic nanoparticles**	Mesoporous silica micro-rods containing IL-2 allow for sustained release of IL-2, and the biocompatible liposomal bilayer coated with anti-CD3 and anti-CD28 enable T cell activation and co-stimulation Protein nanogels containing IL-15SA and anti-CD45 antibodies conjugated to CD8+ T cells release IL-15SA for CAR T cell activation only upon TCR-antigen binding, exhibiting triggered delivery of cargo Polymeric nanoparticles coated with anti-CD3e antibodies and encapsulating CAR plasmid DNA generate selective T cell binding and *in situ* CAR T cell production and activation Mesoporous silica nanoparticles carrying IR780 and coated onto CAR T cells enhance anti-tumor toxicity *via* photothermal therapy	(52, 61, 69, 72)
**Prussian Blue nanoparticles**	Cytotoxic T cells conjugated with Prussian blue nanoparticles mediated improved anti-target cell T cell cytotoxicity with photothermal therapy	(71)

**Figure 1:**
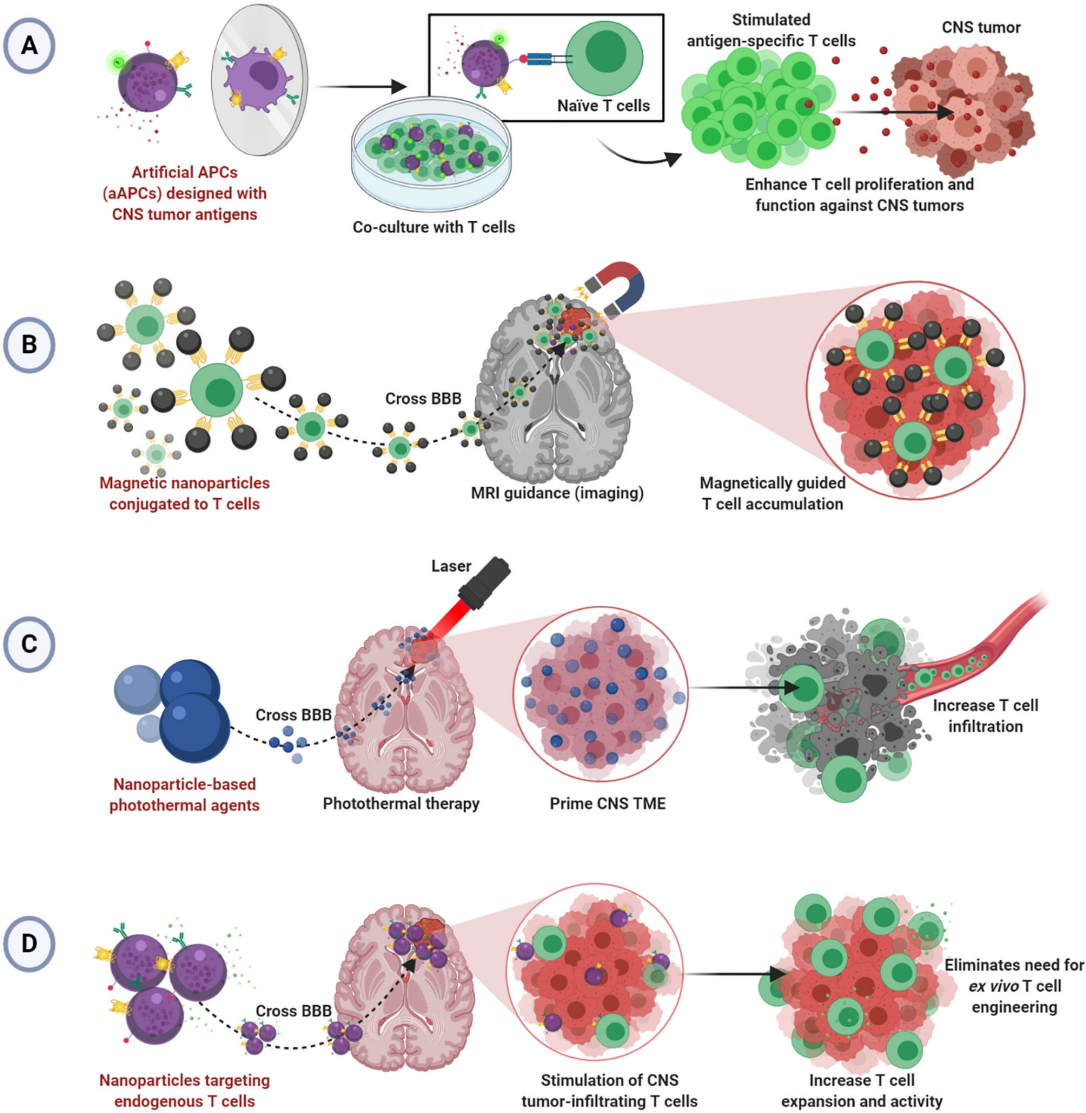
Hypothesized applications of nanoparticle-enhanced T cell therapy for CNS tumors. **(A)** Artificial aAPCs presenting multiple tumor-specific antigens may enable improved *ex vivo* generation of effective CNS tumor-specific T cells. **(B)** Magnetic nanoparticles conjugated to T cells may allow magnetically guided T cell delivery into CNS tumors. **(C)** Nanoparticle-mediated photothermal therapy may allow infiltration of T cells into primed CNS tumors. **(D)** Nanoparticles targeting T cells endogenous to CNS TME may bypass the BBB to enable enhanced T cell function without *ex vivo* T cell engineering.

Although there have been clinical studies demonstrating safety and feasibility of T cell therapy for glioblastoma (2, 73–75), tumors of the CNS remain difficult to treat with adoptive T cell therapy due to limitations typical of solid tumors in addition to the restrictive anatomical barriers that prevent adequate delivery (76, 77). Despite the benefits of using nanoparticles to enhance T cell therapy illustrated in the work described herein, no studies show efficacy in the CNS tumor setting. We hypothesize that the principles of nanoparticles enhancing T cell therapy for solid tumors can be applied successfully to treat brain and CNS tumors. We have summarized our hypothesized applications of nanoparticle-enhanced T cell therapy for CNS tumors in **Figure 1**.

Studies demonstrating improved T cell activation and expansion *via* aAPCs or nanoparticle-based T cell scaffolds prior to adoptive transfer can be refined for application in CNS tumors using CNS tumor-specific antigens. Since aAPCs can be engineered to express CNS tumor antigens, they could seamlessly change disease contexts. Indeed, there are antigen-specific and CAR T cell products to target CNS tumors (78, 79), and several have been investigated clinically (2, 73–75). We propose that aAPC-stimulated T cells may offer a more effective approach to development. Further, aAPCs using multiple antigens, as seen in neoantigen vaccines for glioblastoma (80, 81), could help to target the heterogeneous TME and induce a more complete T cell response (**Figure 1A**). However, because of the anatomical barriers hindering the infiltration of transferred T cells into the brain and spinal cord, we speculate that therapeutic T cells will need to be administered intratumorally in the absence of other TME manipulations or additional functionalities.

Several described studies offer insight into a hypothesized strategy for driving adopted T cell infiltration into CNS tumors. The research using MRI guidance and external magnets to drive magnetosome- or magnetic nanocluster-conjugated T cells into tumors could be applicable (53, 67). There, perhaps a magnetic field could magnetically attract CNS tumor-specific T cells armed with the magnetic nanoparticles as well, thereby overcoming the limitation of poor T cell penetration into tumors (**Figure 1B**). Alternatively, several noted studies critically illuminated a possible strategy for T cell delivery in CNS tumors by photothermal therapy (65, 71, 72). Photothermal therapy could be used to prime the tumor and/or BBB to allow increased vasodilation and T cell infiltration into CNS tumors. Thus, poor T cell infiltration would be mitigated (**Figure 1C**).

Lastly, endogenous T cells in CNS tumors can be exploited by employing methods to target T cells *in situ* as described (69, 70). These strategies rely on re-programming T cells already in the tumors to overcome immunosuppression and/or add functionality. Since nanoparticles could feasibility bypass the BBB to enter the brain and spinal cord, this design is particularly suited to treating CNS tumors (**Figure 1D**). However, efficacy may be limited depending on the number of endogenous T cells in the tumor. Recent work demonstrates the feasibility of BBB-permeable nanoparticles to drive infiltration of T cells into the brain TME, illustrating an interesting potential solution to this issue (82).

We have highlighted the state of the field of nanoparticle-enhanced T cell therapy and commented on the applicability of the principles described herein to treat CNS tumors. The ability of nanoparticles to efficiently activate therapeutic T cells prior to adoptive transfer, create an immunopermissive TME, and add specific targeting capabilities or functionalities, enables the efficacy of adoptive T cell therapy and CAR T cells against cancer. There are several promising avenues for T cell therapy to succeed in CNS tumors, but the anatomical barriers will narrow the applicable options. The research illustrated here suggests that nanotechnology can be key in solving many issues associated with treating CNS tumors with T cell therapy.

## Data Availability Statement

The original contributions presented in the study are included in the article/Supplementary Material. Further inquiries can be directed to the corresponding author.

## Author Contributions

PBB and ES researched and wrote the article. Both authors contributed to the article and approved the submitted version.

## Funding

Funding was provided by the GW Cancer Center, George Washington University, Washington, DC.

## Conflict of Interest

The authors declare that the research was conducted in the absence of any commercial or financial relationships that could be construed as a potential conflict of interest.
